# Trends in all-cause mortality and major causes of death between 2007 and 2018 among patients with diabetes in Taiwan

**DOI:** 10.3389/fendo.2022.984137

**Published:** 2022-08-09

**Authors:** Jun-Sing Wang, Yi-Ling Wu, Horng-Yih Ou, Yi-Sun Yang, Chih-Cheng Hsu, Chii-Min Hwu

**Affiliations:** ^1^ Division of Endocrinology and Metabolism, Department of Internal Medicine, Taichung Veterans General Hospital, Taichung, Taiwan; ^2^ Department of Medicine, School of Medicine, National Yang Ming Chiao Tung University, Taipei, Taiwan; ^3^ College of Medicine, National Chung Hsing University, Taichung, Taiwan; ^4^ Institute of Biomedical Sciences, National Chung Hsing University, Taichung, Taiwan; ^5^ Institute of Population Health Sciences, National Health Research Institute, Miaoli, Taiwan; ^6^ Division of Endocrinology and Metabolism, Department of Internal Medicine, National Cheng Kung University Hospital, Tainan, Taiwan; ^7^ College of Medicine, National Cheng Kung University, Tainan, Taiwan; ^8^ Division of Endocrinology and Metabolism, Department of Internal Medicine, Chung Shan Medical University Hospital, Taichung, Taiwan; ^9^ Institute of Medicine, School of Medicine, Chung Shan Medical University, Taichung, Taiwan; ^10^ National Center for Geriatrics and Welfare Research, National Health Research Institutes, Miaoli, Taiwan; ^11^ Department of Health Services Administration, China Medical University, Taichung, Taiwan; ^12^ Department of Family Medicine, Min-Sheng General Hospital, Taoyuan, Taiwan; ^13^ Section of Endocrinology and Metabolism, Department of Medicine, Taipei Veterans General Hospital, Taipei, Taiwan

**Keywords:** diabetes, mortality, survival, cancer, heart disease

## Abstract

Optimal control of diabetes and relevant risk factors substantially reduce the risks of chronic complications and mortality. We investigated all-cause mortality rate and major causes of death between 2007 and 2018 in patients with diabetes in Taiwan. This study was conducted using data from Taiwan National Health Insurance Research Database. We selected patients with diabetes diagnosed between 2007 and 2017 (grouped according to the year of diabetes diagnosis 2007-2010 vs. 2011-2017). Information on mortality and causes of death by the end of 2018 was confirmed through linking to the National Death Registry. Standardized mortality rate (SMR) were calculated by weighting the World Health Organization (WHO) standard population (WHO 2000-2025). More than 2.7 million of patients with diabetes were analyzed and a total of 566121 deaths were identified. Overall, the SMR was 11.72 per 1000 person-years. Patients with diabetes diagnosed in 2011-2017 had a lower SMR (8.42 vs. 12.92 per 1000 person-years) than those diagnosed in 2007-2010. Similar finding were noted regarding the major causes of death (cancer, diabetes, heart disease, hypertensive disease, and cerebrovascular disease). Compared with patients who were diagnosed in 2008-2010, those who were diagnosed in 2011-2014 and 2015-2018 had a higher 3-year survival rate (0.9356 vs. 0.9438 vs. 0.946, log-rank test p<0.001) after the diagnosis of diabetes. Patients who were diagnosed with diabetes after 2011 had a lower rate of all-cause mortality and major causes of death, compared with those who were diagnosed before 2010 in Taiwan.

## Introduction

The continuous increase in the number of patients with diabetes (1–3) makes the disease become a major healthcare burden. According to a recent estimate (1), the global prevalence of adult people with diabetes was 536.6 million in 2021. The number was estimated to be 783.2 million in 2045 (1). Patients with diabetes are associated with a higher risk of micro- and macro-vascular complications (3–6), both of which lead to morbidities and mortality (5–7). There were 6.7 million of deaths due to diabetes globally in 2021, and total health expenditure due to diabetes was 966 billion USD (1).

Optimal control of diabetes and relevant risk factors could substantially reduce the risks of chronic complications and mortality (8–10). Since the launch of National Health Insurance (NHI) program in 1995 and the initiation of diabetes pay-for-performance (P4P) program in 2001 (11, 12), the outcomes of patients with diabetes in Taiwan have been improved overtime (13–15). The all-cause mortality rate in patients with diabetes declined between 2000 and 2014 (13, 14). Nevertheless, the healthcare quality for diabetes in Taiwan was rated as suboptimal in a recent report (16).

In this study, we aimed to update the rates of all-cause mortality and major causes of death in patients with diabetes in Taiwan. We investigated the mortality rates among patients diagnosed in different time periods to examine the differences in outcomes.

## Materials and methods

### Database and ethical approval

This study was conducted using data from Taiwan National Health Insurance Research Database (NHIRD). Since the launch of the NHI program in 1995, more than 99% of the inhabitants in Taiwan are covered by the program which represents an important source for healthcare quality assessments and researches. De-identified data were released by the Health and Welfare Data Science Center for analyses and research use. This study was conducted in accordance with the Declaration of Helsinki. The study protocol was approved by the Research Ethics Committee of the National Health Research Institute (approval number: EC1020408-E) prior to study procedures.

### Study population and outcomes

We selected patients with diabetes diagnosed between 2007 and 2017 for analyses. Patents were considered as having diabetes if they had 3 or more outpatient clinic visits or one hospital admission with the diagnosis of diabetes in 1 year (17). The diagnosis of diabetes was based on the International Classification of Diseases (ICD), 9^th^ and 10^th^ Revision, Clinical Modification. For ICD-9-CM, the codes for diabetes diagnosis were 250.x. For ICD-10-CM, the codes were E0-E14. Patients were excluded from the analyses if they had missing information on sex or date of birth. Patients were grouped according to the year of diabetes diagnosis (2007-2010 vs. 2011-2017). Information on mortality and causes of death by the end of 2018 was confirmed through linking to the National Death Registry. To avoid confounding effect of diabetes duration on risk of mortality, we also examined the survival rate annually after diabetes diagnosed in different periods (2008-2010 vs. 2011-2014 vs. 2015-2018).

### Statistical analyses

All the statistical analyses were performed using SAS version 9.4 (SAS Institute, Cary, NC, USA). We examined the crude mortality rate (expressed as per 1000 person-years) for all-cause mortality and top 5 causes of death in the study population, and in subgroups by sex, age, and year of diabetes diagnosis. We calculated standardized mortality rate (SMR) (expressed as per 1000 person-years) by weighting the World Health Organization (WHO) standard population (WHO 2000-2025). The annual survival rates after diabetes diagnosis were compared among patients diagnosed in different periods (2008-2010 vs. 2011-2014 vs. 2015-2018).

## Results

**Table 1** shows all-cause mortality rate in patients with diabetes between 2007 and 2017. Overall, more than 2.7 million of patients with diabetes were analyzed and a total of 566121 deaths were identified. The crude mortality rate was 29.64 per 1000 person-years, while the SMR was 11.72 per 1000 person-years. The mortality rate in men was higher than in women (14.57 vs. 8.82 per 1000 person-years), and the findings were similar across all age subgroups. **Table 2** shows all-cause mortality rate by year of diabetes diagnosis (2007-2010 vs. 2011-2017). Overall, patients with diabetes diagnosed in 2011-2017 had a lower crude mortality rate (19.72 vs. 32.89 per 1000 person-years) and SMR (8.42 vs. 12.92 per 1000 person-years) than those diagnosed in 2007-2010. The findings were consistent in men and in women.

**Table 1:** All-cause mortality rates in patients with diabetes between 2007 and 2017 (follow up to the end of 2018).

	Number of patients	Number of death	Crude mortality rate	Standardized mortality rate
All	2773077	566121	29.64	11.72
Men	1452765	313979	32.22	14.57
Women	1320312	252142	26.96	8.82
All: by age of diabetes diagnosis				
≤ 40 years	199065	7865	5.98	—
41-50 years	416920	30218	10.10	—
51-60 years	793631	82974	14.21	—
61-70 years	696220	127848	26.23	—
≥ 71 years	667241	317216	77.72	—
Men: by age of diabetes diagnosis				
≤ 40 years	122627	5985	7.45	—
41-50 years	265715	23110	12.36	—
51-60 years	429756	56118	18.18	—
61-70 years	333733	72874	32.70	—
≥ 71 years	300934	155892	88.72	—
Women: by age of diabetes diagnosis				
≤ 40 years	76438	1880	3.68	—
41-50 years	151205	7108	6.34	—
51-60 years	363875	26856	9.76	—
61-70 years	362487	54974	20.79	—
≥ 71 years	366307	161324	69.40	—

Mortality rate is numbers per 1000 person-years. The standardized mortality rate was calculated by weighting the World Health Organization (WHO) standard population (WHO 2000-2025).

**Table 2:** All-cause mortality rates in diabetes patients by year of diabetes diagnosis (2007-2010 versus 2011-2017, follow up to 2018).

	Number of patients	Number of death	Crude mortality rate	Standardized mortality rate
All				
2007-2010	1542933	473215	32.89	12.92
2011-2017	1230144	92906	19.72	8.42
Men				
2007-2010	791898	257172	35.45	15.93
2011-2017	660867	56807	22.79	10.99
Women				
2007-2010	751035	216043	30.29	9.85
2011-2017	569277	36099	16.27	5.80

Mortality rates are numbers per 1000 person-years. The standardized mortality rate was calculated by weighting the World Health Organization (WHO) standard population (WHO 2000-2025).

The major causes of death in patients with diabetes between 2007 and 2017 are shown in **Table 3**. The top 5 causes of death accounted for nearly 70% (394664/566121, 69.7%) of the total number of deaths. Malignancy (23.2%) was the leading cause of death, followed by diabetes (16.7%), heart disease (11.6%), hypertensive disease (11.3%), and cerebrovascular disease (7.0%). Men had a higher mortality rate than women in all the 5 causes of death, and the findings were consistent across all age subgroups.

**Table 3:** Major causes of death in patients with diabetes between 2007 and 2017 (follow up to the end of 2018).

	Cancer		Diabetes		Heart Disease		Hypertensive disease		Cerebrovascular disease		
	Number	MR (SMR)	Number	MR (SMR)	Number	MR (SMR)	Number	MR (SMR)	Number	MR (SMR)	
All	131177	6.87 (2.47)	94468	4.95 (2.07)	65426	3.43 (1.28)	63863	3.34 (1.26)	39730	2.08 (0.69)	
Men	79321	8.14 (3.12)	46471	4.77 (2.37)	35835	3.68 (1.62)	35000	3.59 (1.59)	21844	2.24 (0.83)	
Women	51856	5.55 (1.81)	47997	5.13 (1.76)	29591	3.16 (0.93)	28863	3.09 (0.91)	17886	1.91 (0.55)	
All: by age										
≤ 40 years	1102	0.84	1563	1.19	800	0.61	793	0.60	298	0.23	
41-50 years	7684	2.57	4888	1.64	3164	1.06	3128	1.05	1563	0.52	
51-60 years	26039	4.46	13177	2.26	9006	1.54	8852	1.52	4900	0.84	
61-70 years	37018	7.60	21794	4.47	14020	2.88	13737	2.82	8619	1.77	
≥ 71 years	59334	14.54	53046	13.00	38436	9.42	37353	9.15	24350	5.97	
Men: by age										
≤ 40 years	795	0.99	1136	1.41	641	0.80	637	0.79	204	0.25	
41-50 years	5728	3.06	3550	1.90	2468	1.32	2438	1.30	1191	0.64	
51-60 years	17578	5.69	8338	2.70	6419	2.08	6308	2.04	3494	1.13	
61-70 years	22343	10.03	11280	5.06	8079	3.63	7910	3.55	5225	2.34	
≥ 71 years	32877	18.71	22167	12.62	18228	10.37	17707	10.08	11730	6.68	
Women: by age										
≤ 40 years	307	0.60	427	0.84	159	0.31	156	0.31	94	0.18	
41-50 years	1956	1.75	1338	1.19	696	0.62	690	0.62	372	0.33	
51-60 years	8461	3.08	4839	1.76	2587	0.94	2544	0.92	1406	0.51	
61-70 years	14675	5.55	10514	3.98	5941	2.25	5827	2.20	3394	1.28	
≥ 71 years	26457	11.38	30879	13.29	20208	8.69	19646	8.45	12620	5.43	

MR, mortality rate (per 1000 person-years). SMR, standardized mortality rate (per 1000 person-years). The SMR was calculated by weighting the World Health Organization (WHO) standard population (WHO 2000-2025).

**Table 4** shows the major causes of death by year of diabetes diagnosis (2007-2010 vs. 2011-2017). With respect to the top 5 causes of death, patients with diabetes diagnosed in 2011-2017 had a lower crude mortality rate and SMR than those diagnosed in 2007-2010. For example, the crude mortality rate and SMR for cancer (follow up to the end of 2018) in patients with diabetes diagnosed between 2007 and 2010 were 7.26 and 2.60 (per 1000 person-years), respectively. The respective rates in those diagnosed between 2011 and 2017 were 5.66 and 2.12 (per 1000-person years). The findings were consistent in men and in women.

**Table 4:** Major causes of death in patients with diabetes by year of diabetes diagnosis (2007-2010 versus 2011-2017, follow up to the end of 2018).

	Cancer		Diabetes		Heart Disease		Hypertensive disease		Cerebrovascular disease	
	Number	MR (SMR)	Number	MR (SMR)	Number	MR (SMR)	Number	MR (SMR)	Number	MR (SMR)
All										
2007-2010	104498	7.26 (2.60)	85612	5.95 (2.48)	54914	3.82 (1.40)	53352	3.71 (1.37)	33284	2.31 (0.77)
2011-2017	26679	5.66 (2.12)	8856	1.88 (0.93)	10512	2.23 (0.93)	10511	2.23 (0.93)	6446	1.37 (0.48)
Men										
2007-2010	62381	8.60 (3.29)	41369	5.70 (2.82)	29459	4.06 (1.75)	28624	3.95 (1.71)	18030	2.49 (0.91)
2011-2017	16940	6.80 (2.68)	5102	2.05 (1.16)	6376	2.56 (1.27)	6376	2.56 (1.27)	3814	1.53 (0.59)
Women										
2007-2010	42117	5.90 (1.90)	44243	6.20 (2.13)	25455	3.57 (1.05)	24728	3.47 (1.02)	15254	2.14 (0.62)
2011-2017	9739	4.39 (1.55)	3754	1.69 (0.69)	4136	1.86 (0.58)	4135	1.86 (0.58)	2632	1.19 (0.37)

MR, mortality rate (per 1000 person-years). SMR, standardized mortality rate (per 1000 person-years). The SMR was calculated by weighting the World Health Organization (WHO) standard population (WHO 2000-2025).

**Figure 1:**
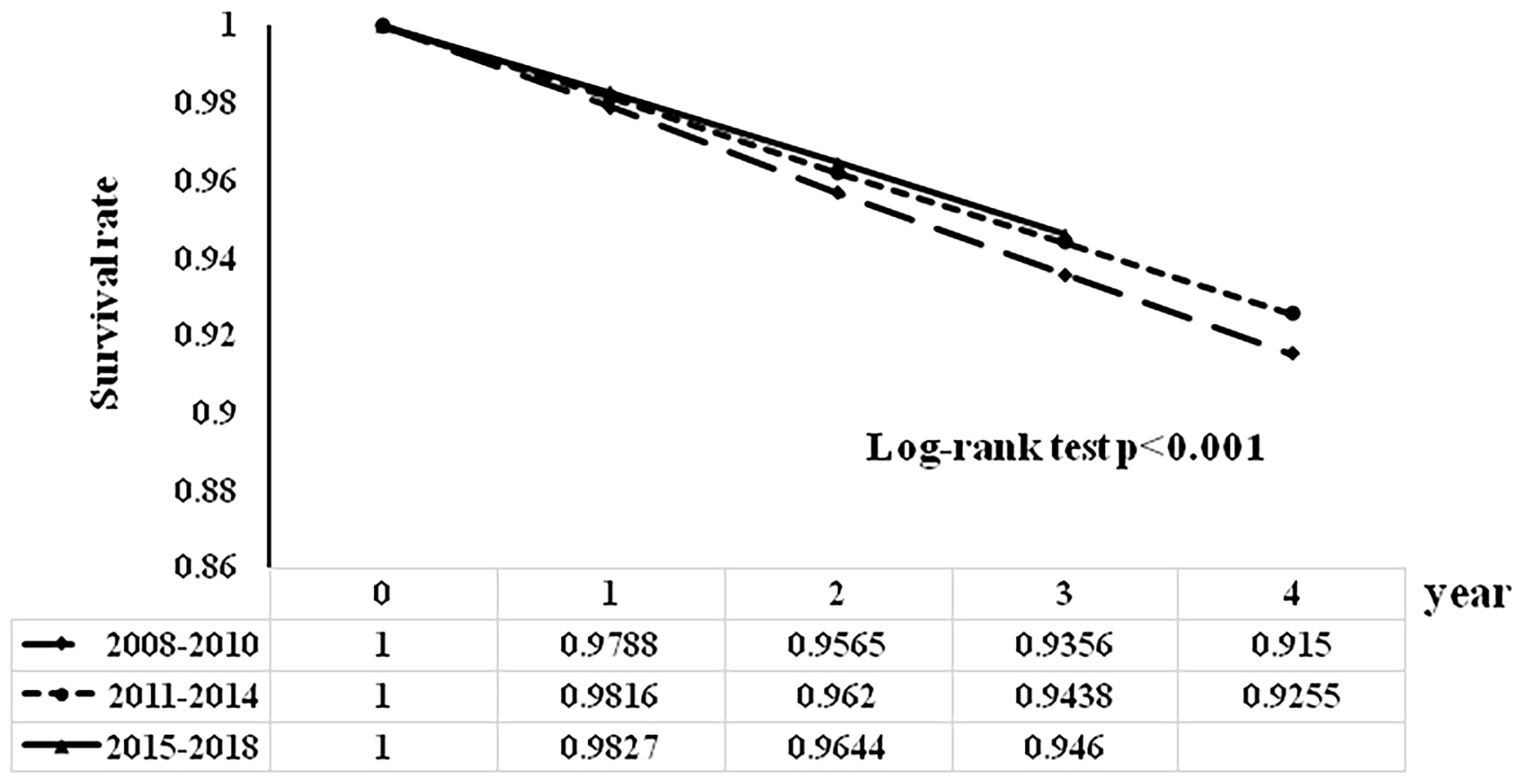
Survival rates after the diagnosis of diabetes by year of diagnosis.

The survival rates after the diagnosis of diabetes according to year of diagnosis are shown in **Figure 1**. Compared with patients who were diagnosed in 2008-2010, those who were diagnosed in 2011-2014 and 2015-2018 had a higher 3-year survival rate (0.9356 vs. 0.9438 vs. 0.946, log-rank test p<0.001) after the diagnosis of diabetes.

## Discussion

In this study, we investigated all-cause mortality rate and major causes of death between 2007 and 2018 in patients with diabetes in Taiwan. We demonstrated that patients with diabetes diagnosed in 2011-2017 had a lower all-cause mortality rate (SMR 8.42 vs. 12.92 per 1000 person-years) than those diagnosed in 2007-2010. The findings were consistent in men and in women. Similar results were noted regarding the major causes of death (cancer, diabetes, heart disease, hypertensive disease, and cerebrovascular disease). Our results suggested an outcome improvement in diabetes patients during 2007-2018 in Taiwan.

All-cause mortality rate in patients with diabetes has declined in the past decades (6, 18–20). In a recent report (21), all-cause mortality rates among people with diabetes in high-income countries continue to decline between 1995 and 2016. The improvement in patients’ survival may be explained by optimal control of risk factors (8–10, 22) and the use of guidelines-recommended treatment (23, 24). In patients with diabetes in Taiwan, the control rates of glycemia, blood pressure, and low-density lipoprotein cholesterol (25–27), as well as the use of statins (28), continuously increased during our study period. Furthermore, the promotion of pay-for-performance program for diabetes care (29, 30) may also contribute to the reduction of all-cause mortality rate in our patients. The standardized mortality rate in our study (8-13 per 1000 person-years) was similar to the finding in a previous study (~15 per 1000 person-years) (31), which also revealed a decline in all-cause mortality rate in patients with diabetes between 1996 and 2009. Advances in the availability and performance of technology, developments of new drugs, pleiotropic effects of guidelines-recommended treatment (32), and technology-assisted facilitation of disease screening and patients’ follow-up (33, 34) may all contribute to the improvements in outcomes.

The decline in all-cause mortality rate was consistently observed in the major causes of deaths in our study population. The SMR (per 1000 person-years) of cancer mortality in 2007-2010 and 2011-2017 was 7.26 and 5.66, respectively (**Table 4**). Similar declines in the other causes of deaths (diabetes, heart disease, hypertensive disease, and cerebrovascular disease) were observed. Since cancer and cardiovascular disease share similar risk factors (35), it may not be surprised that improvement in diabetes care, the use of guidelines-recommended treatment (such as metformin) (32, 36), and optimal control of risk factors lead to a decline in rates of cancer and cardiovascular mortality. However, patients with diabetes remain at a higher risk of mortality compared with patients without diabetes (6, 37, 38). In 2018, the crude mortality rate and SMR of the Taiwan population were 7.33 and 4.15 per 1000 person-years, respectively (https://dep.mohw.gov.tw/DOS/lp-5069-113.html ). The respective rates of our study population were 29.64 and 11.72 per 1000 person-years (**Table 1**). According to our findings, cancer and cardiovascular diseases remain the major causes of death in patients with diabetes in Taiwan. Inflammatory pathways associated with adipose tissue (39, 40) have been associated with an increase in oxidative and cellular stress (39, 40). These may contribute to the chronic inflammation and unfavorable outcomes in patients with diabetes and cardiovascular diseases (41) or heart failure (42, 43). Hence, continuous efforts are required to promote health care for patients with diabetes to improve their outcomes despite our encouraging findings.

There are several limitations in this study. First, we did not have data on glycemia, blood pressure, and lipid profiles. These are important factors related to diabetes care and risk of mortality. Second, we did not have data on relevant medications, such as glucose- and blood pressure-lowering drugs, as well as statins. The use of some guidelines-recommended drugs (e.g. statins) may help reduce mortality risk in patients with diabetes (23, 24). Unfortunately, this cannot be examined in this study. Final, relevant information on lifestyles, such as smoking, alcohol consumption, and physical activity, are lacking. All these issues may influence patients’ survival and should be addressed in future studies. Despite the aforementioned limitations, our findings from a large database may provide insight on diabetes care program and health promotion policy.

In conclusion, we demonstrated an outcome improvement in patients with diabetes in Taiwan. Patients who were diagnosed with diabetes after 2011 had a lower rate of all-cause mortality and major causes of death, compared with those who were diagnosed before 2010. These promising findings may have implications for healthcare systems.

## Data availability statement

The datasets presented in this article are not readily available due to privacy/ethical restrictions. Requests to access the datasets should be directed to C-CH, https://cch@nhri.edu.tw.

## Ethics statement

The studies involving human participants were reviewed and approved by The Research Ethics Committee of the National Health Research Institute. Written informed consent for participation was not required for this study in accordance with the national legislation and the institutional requirements.

## Author contributions

J-SW, H-YO, Y-SY, and C-MH contributed to conception and design of the study. Y-LW, C-CH, and C-MH organized the database. Y-LW and C-CH performed the statistical analysis. J-SW and Y-LW wrote the first draft of the manuscript. H-YO, Y-SY, C-CH, and C-MH reviewed and edited the manuscript. All authors contributed to manuscript revision, read, and approved the submitted version.

## Funding

This research was funded by the Diabetes Association of the Republic of China [grant number DAROC2021ATLAS-0001, 2021] and the Taiwanese Association of Diabetes Educators [grant number TADE-2021-RES-01, 2021]. The sponsors had no role in the design, execution, interpretation, or writing of the study.

## Acknowledgments

We thank the Health and Welfare Data Science Center for providing data and Institute of Population Health Sciences, National Health Research Institutes for performing analysis.

## Conflict of interest

The authors declare that the research was conducted in the absence of any commercial or financial relationships that could be construed as a potential conflict of interest.

## Publisher’s note

All claims expressed in this article are solely those of the authors and do not necessarily represent those of their affiliated organizations, or those of the publisher, the editors and the reviewers. Any product that may be evaluated in this article, or claim that may be made by its manufacturer, is not guaranteed or endorsed by the publisher.
